# Sodium-myoinositol cotransporter-1 downstream of m6A methyltransferase WTAP exerts a potential carcinogenicity in diffuse large B-cell lymphoma progression

**DOI:** 10.1186/s12967-025-07303-7

**Published:** 2025-11-18

**Authors:** Xinyang Li, Xiaowei He, Ying Sun, Wei Yang

**Affiliations:** Department of Hematology, Shengjing Hospital of China Medical University, 36# Sanhao Street, Shenyang, China

**Keywords:** SMIT1, Diffuse large B-cell lymphoma, N6-methyladenosine, WTAP

## Abstract

**Background:**

Diffuse large B-cell lymphoma (DLBCL) is the most common subtype of aggressive non-Hodgkin lymphoma, contributing significantly to global health and economic challenges. Sodium-myoinositol cotransporter-1 (SMIT1) acts as an oncogene in different types of cancer. This study aimed to explore [1] the role of SMIT1 in DLBCL development, and [2] whether N6-Methyladenosine (m6A) modifications were responsible for high SMIT1 expression within DLBCL tissues.

**Methods:**

Expression of SMIT1 was modulated by a eukaryotic SMIT1-expressing plasmid or a plasmid specific to SMIT1-targeting shRNA. The impacts of SMIT1 overexpression or knockdown on DLBCL cell proliferation, cell cycle, and apoptosis were evaluated using cell-counting-kit-8, flow cytometry and western blot assays. A DLBCL cell-derived tumor xenograft was established to further assess the tumorigenicity of SMIT1. qPCR, RIP-qPCR, MeRIP-qPCR, dual luciferase reporter and western blot assays were employed to explore whether high SMIT1 expression was associated with WTAP/YTHDF1-mediated m6A modifications.

**Results:**

Bioinformatics analysis showed that high SMIT1 expression in DLBCL was positively associated with poor prognosis, survival-related markers and m6A methyltransferase Wilms tumor 1-associated protein (WTAP). SMIT1 overexpression supported proliferation and cell cycle progression of DLBCL cells, while its depletion induced proliferation suppression, G1-S phase arrest and apoptosis of DLBCL cells. Decreased myo-inositol, phosphatidylinositol 4,5-bisphosphate (PIP2) and phosphatidylinositol 3,4,5-trisphosphate (PIP3) contents and AKT phosphorylation level were observed after SMIT1 silencing, yet increased after SMIT1 overexpression in DLBCL cells. In vivo, SMIT1 silencing delayed tumor growth and induced AKT inactivation. SMIT1 silencing-induced anti-DLBCL role was partly weakened by the addition of AKT agonist SC-79. Furthermore, we found that upregulation of WTAP enhanced the SMIT1 m6A and mRNA levels. WTAP-regulated SMIT1 m6A was recognized and stabilized by YTH N6-Methyladenosine RNA Binding Protein F1 (YTHDF1) m6A reader.

**Conclusions:**

Our study uncovers a novel oncogenic axis in DLBCL, where SMIT1’s carcinogenic potential is epigenetically modulated by WTAP/YTHDF1-mediated m6A methylation.

**Supplementary Information:**

The online version contains supplementary material available at 10.1186/s12967-025-07303-7.

## Background

Diffuse large B-cell lymphoma (DLBCL) is the most common subtype of aggressive non-Hodgkin lymphoma (NHL), accounting for 30–60% of NHL cases [1]. Patients without treatment will typically die weeks or months after diagnosis. Significant advances in the treatment of DLBCL have been made with the advent of rituximab-based first-line immunochemotherapy [2]. However, 30–40% of patients still suffer from relapsed or refractory disease [3]. A large proportion of them are not candidates for second-line transplantation/intensive therapy. Therefore, it is imperative to further elucidate the molecular pathogenesis of DLBCL in order to develop novel therapeutic strategies for advanced and recurrent patients.

Myo-inositol is an abundant carbocyclic sugar alcohol in the human body. It is a head group of phosphoinositides, which are key signaling molecules including phosphatidylinositol 4,5-bisphosphate [PI [4, 5]P2, PIP2] and phosphatidylinositol 3,4,5-trisphosphate [PI [3–5]P3, PIP3] [4]. In this lipid context, the phosphoinositides are dynamically modulated by kinases [eg, phosphatidylinositol-3 kinase (PI3K)] and phosphatases [eg, Tumor suppressors phosphatase and tensin homolog (PTEN)] to control the AKT/PKB (protein kinase B) signaling [5]. D’Oria et al. demonstrated that exogenous administration of myo-inositol activated the AKT signaling in human endothelial cells [6]. Inactivation of the AKT signaling has been proven to delay DLBCL progression [7, 8]. Thus, regulating inositol levels and affecting phosphatidylinositol signaling may be a potential approach for the treatment of DLBCL—a possibility that warrants further investigation.

Sodium-myoinositol cotransporter-1 (SMIT1), the third member of the solute carrier transporter family 5 (SLC5A3), has been mainly studied in the brain, kidney and heart where it mediates the transport of extracellular myo-inositol into the cell interior [9]. Accumulating evidence highlights SMIT1 as a tumor-promoting factor in multiple malignancies, such as glioblastoma, non-small cell lung cancer (NSCLC), and cervical cancer [10–12]. It is reported that knockdown of SMIT1 delays tumor growth in acute myeloid leukemia by decreasing intracellular myo-inositol level [13]. Data from the GEPIA public database revealed that SMIT1 was highly expressed in DLBCL tissues. In view of these findings, high expression of SMIT1 might be involved in the progression of DLBCL due to its role in myo-inositol transport.

Abundant evidence has demonstrated that N6-methyladenosine (m6A) modification of gene transcripts plays critical roles in DLBCL. This modification is a dynamic and reversible process that is controlled by “writers” (methyltransferases), “erasers” (demethylases) and “readers” (effector proteins). A classical complex of writers, including methyltransferase-like 3 (METTL3), methyltransferase-like 14 (METTL14) and Wilms tumor 1-associated protein (WTAP), drives m6A subtype formation. The “readers” containing YTH domains can specifically bind to “writers”-modified RNA to regulate mRNA splicing, degradation, stability, and translation. Here, we attempted to understand whether m6A modification of SMIT1 was responsible for its upregulation in DLBCL. In addition, the tumorigenicity and potential mechanism of SMIT1 in DLBCL were evaluated using two DLBCL cell lines and cell-derived tumor xenografts.

## Methods

### Acquisition of public data

The bioinformatics tool GEPIA (version 2.0, http://gepia.cancer-pku.cn/index.html) was used to analyze differential expression of SMIT1 between DLBCL and normal tissues from the freely accessible data banks Genotype-Tissue Expression project (GTEx) and The Cancer Genome Atlas (TCGA). Similarly, we obtained the gene expression matrix of DLBCL patients in the GSE32018 microarray dataset from the Gene Expression Omnibus (GEO) database (https://www.ncbi.nlm.nih.gov/geo/).

### Data collection, pre-processing, and identification of differentially upregulated genes

The criteria of a 1.5-fold change and adj.*p* < 0.05 were applied to screen the differentially upregulated genes (coUps). Volcano plots and heatmap of the coUps were drawn using R v4.3.2 ggplot2 v3.5.0 and pheatmap v1.0.12. The Annotation, Visualization and Integrated Discovery (DAVID) resource was used for gene ontology (GO) enrichment analysis of coUps, which included biological processes (BP), molecular functions (MF) and cellular components (CC).

### Patients and specimens

Patients who were diagnosed with DLBCL gave informed consent and were enrolled in this study, and paraffin-embedded specimens were collected. This study was approved by the Medical Ethical Committee of Shengjing Hospital of China Medical University (approval ID: 2024PS1336K). The detailed clinical characteristics of the patients are listed in Table S1.

### Cell culture

Human DLBCL cell lines (U2932 and OCI-Ly10) and HEK293T cells were gifted from iCell (Shanghai, China). The U2932 (accession number: CVCL_1896) and OCI-Ly10 (accession number: CVCL_8795) cell lines have undergone identity verification via the Cellosaurus database (https://www.cellosaurus.org/), with both explicitly confirmed to belong to the activated B-cell-like (ABC) subtype of DLBCL. U2932 and HEK293T cells were cultured in RPMI-1640 complete medium (GIBCO, Carlsbad, CA, USA, 31800) supplemented with 10% fetal bovine serum (FBS) (Sangon, Shanghai, China, B548117) and 1% penicillin-streptomycin solution (P/S), while OCI-Ly10 cells with 20% FBS and 1% P/S. All cells were maintained in a humidified cell incubator at 37 °C with an atmosphere of 5% CO_2_.

### Plasmid transfection and construction of stable cell lines

*Homo* SMIT1 CDS (NM_006933) was inserted into pCDNA3.1 (+) plasmid to induce SMIT1 overexpression. Two shRNAs targeting *homo* SMIT1 mRNA were inserted into pRNA-H1.1/Neo plasmid to silence SMIT1. The plasmids were transiently transfected into U2932 and OCI-Ly10 cells using Lipofectamine 3000 (Invitrogen, Carlsbad, CA, USA, L3000-008) according to the manufacturer’s instructions. Cells were then maintained in a humidified cell incubator at 37 °C with 5% CO_2_. At 24 h after transfection, the cells were cultured in medium containing G418. Culture media were changed every 2–3 days for 2–3 weeks. G418-resistant clones were picked and amplified for stable expression.

To determine whether SMIT1 upregulation in DLBCL is attributed to its m6A modification, the m6A writer WTAP overexpression pCDNA3.1 (+) plasmid and WTAP targeted RNA interference recombinant pRNA-H1.1/Neo plasmids were transiently transfected into ABC-DLBCL cells (U2932 and OCI-Ly10). The pRNA-H1.1/Neo plasmids of RNA interference for YTHDF1/2/3 were constructed and co-transfected into ABC-DLBCL cells with the pCDNA3.1 (+)-WTAP plasmid. At 48 h after transfection, the cells were used for follow-up experiments. The sequences for shRNA or siRNA were listed in Table S2.

### Cell treatment

To determine the effect of SMIT1 on the PI3K-AKT pathway, the SMIT1 stably expressed ABC-DLBCL cells (U2932 and OCI-Ly10) were treated with the AKT agonist SC-79 (5 µM; Macklin, Shanghai, China; S873794) or PI3Kα/δ inhibitor Copanlisib (1 µM; Macklin, C873222) for 24 h, respectively.

### Cell-counting-kit-8 (CCK8) assay

Cells were seeded into 96-well plates with five replicate wells in each group (5 × 10^3^ cells/well). After being incubated for 0, 24, 48 and 72 h, 10 µl of CCK8 solution (Biosharp Technology Inc., Hefei, China, BS350A) was added to each well. The absorbance at 450 nm was measured after 2 h of incubation to assess cell viability.

### E-Click 5-ethynyl-2-deoxyuridine (EdU) flow cytometry assay

EdU Cell Proliferation assay was performed using an E-Click plus EdU FITC Flow Cytometry Assay Kit according to the manufacturer’s instructions (Elabscience, Houston, TX, USA, E-CK-A370). Cells were seeded in 6-well plates at a density of 2 × 10^5^ cells each well. Cells were stained with 10 µM EdU solution for 2 h, followed by incubation in 4% polyformaldehyde for 15 min at room temperature protected from light. The fixed cells were treated with 500 µl of PBS plus 1% saponin for 20 min and then labelled with 500 µl of Click-iT reaction cocktail in the dark for 30 min. The newly synthesized DNA was measured using NovoCyte flow cytometry (Agilent, Santa Clara, CA, USA) to assess cell proliferation.

### Flow cytometry-based cell cycle and apoptosis assays

Cell cycle progression was assessed by measuring the DNA fragment staining with propidium iodide (PI) using a Cell Cycle Assay Kit, according to the manufacturer’s instructions (KeyGen Biotech., Nanjing, China, KGA511). Cells were seeded in 6-well plates at a density of 5 × 10^5^ cells per well. Cells were kept in 70% cold ethanol at 4 °C overnight to enhance cell permeability. The fixed cells were then stained with 500 µl of PI/RNase A solution in the dark for 30 min. The distribution (%) of each cell cycle phase was analyzed immediately by NovoCyte flow cytometry.

Cell apoptosis was evaluated using an Annexin V-FITC/PI Apoptosis Detection Kit, according to the manufacturer’s protocol (Biosharp Technology Inc., BL110A). Cells were seeded in 6-well plates at a density of 2 × 10^5^ cells per well. The cells were stored in 100 µl of binding buffer, after which stained with 5µl of AnnexinV-FITC solution for 15 min and 10 µl of PI solution for 5 min at room temperature, protected from light. After supplementing 400 µl of binding buffer, apoptotic cells were measured via NovoCyte flow cytometry. Apoptosis rate (%) was calculated by the sum of early apoptotic cells (AnnexinV-negative/PI-positive) and late apoptotic cells (AnnexinV-positive/PI-positive).

### In vivo tumorigenicity assay

NOD-SCID mice (6-week-old, half male and half female) were kept in a standard environment at a temperature of 25 ± 1 C and 45–55% relative humidity with a 12:12 h light-dark cycle for one week before experimentation. All animals had free access to food and water. The animal experiments were conducted in strict accordance with the Guide for the Care and Use of Laboratory Animals Eighth Edition, and approved by the Institutional Animal Care and Use Committee of Shengjing Hospital of China Medical University (approval ID: 2024PS1337K). The mice were subcutaneously injected with U2932 cells stably overexpressing SMIT1 or stably expressing shRNA targeting SMIT1 (4 × 10^6^ cells). Tumor volume was measured every four days. Twenty-four days after the implantation, the mice were killed and tumor xenografts were removed, photographed and weighed. Tumor volume (V) was calculated using a caliper by measuring the tumor length (L) and tumor width (W), where V = L × W×0.5. The maximal tumor size did not exceed 17 mm.

### Immunohistochemistry (IHC)

Tumors were fixed, paraffin-embedded and cut into 5-µm sections. After being deparaffinized and rehydrated, the sections were incubated in sub-boiling heat citric acid/ sodium citrate buffer for 10 min to unmask antigenic epitope. When cooled naturally to room temperature, the sections were treated with 3% H2O2 for 15 min to eliminate endogenous peroxidase activity. Non-specific reactions with cellular proteins were blocked with 1% bovine serum albumin (BSA) at room temperature for 15 min. Subsequently, the sections were incubated with anti-Ki67 polyclonal rabbit antibody (1:200 dilution, Proteintech Group Inc., Rosemont, IL, USA, 27309-1-AP) or anti-phospho-AKT-S473 monoclonal rabbit antibody (1:50 dilution, ABclonal, AP1208) at 4°C overnight, followed by the incubation of horseradish peroxidase (HRP)-conjugated goat anti-rabbit IgG secondary antibody (1:500 dilution, ThermoFisher SCIENTIFIC, Pittsburgh, PA, USA, 31460) for 1 h at 37°C. The sections were developed in 3,3’-Diaminobenzidine solution and counterstained with hematoxylin for 3 min. After being dehydrated, the sections were mounted with neutral balsam medium, followed by microscopic examination (400×, Olympus Corporation, Tokyo, Japan).

### TdT-mediated dUTP nick end labeling (TUNEL) apoptosis detection

TUNEL fluorescence staining was performed using an In Situ Cell Death Detection Kit according to the manufacturer’s instructions (Roche Products, Inc., Nutley, NJ, USA, 12156792910). The deparaffinized and rehydrated tumor sections were permeabilized in 50 µl of 0.1% Triton X-100 solution at room temperature for 8 min. Then, the sections were labelled with 50 µl of TUNEL reaction solution for 1 h at 37°C, protected from light. The sections were counterstained with 4’,6-diamidino-2-phenylindole (DAPI) for 5 min in the dark to visualize cell nucleus. Finally, fluorescence images were captured under the Olympus microscope (400×).

### Detection of myo-inositol

The myo-inositol Assay Kit (Megazyme, Bray, Wicklow, Ireland, K-INOSL) was an enzymatic UV method for the specific measurement and analysis of myo-inositol in DLBCL cells. Cells were suspended in PBS and lysed by ultrasound. Subsequently, myo-inositol content in cell suspension was measured using the K-INOSL according to the manufacturer’s instructions.

### ELISA for PIP2 and PIP3

The amount of Phosphatidylinositol 4,5-bisphosphate (PI [4, 5]P2, PIP2) produced in DLBCL cells was detected using a PI [4, 5]P2 Mass ELISA Assay Kit following the manufacturer’s recommendations (Echelon Biosciences, Salt Lake City, Utah, USA, K-4500). For detection of PIP3 in DLBCL cells, a Cloud-Clone Corp. #CEG856Ge EAKIT (Houston, TX, USA) was applied according to the manufacturer’s instructions.

### Quantitative real-time PCR (qPCR)

Total RNA was extracted from cells using TRIpure reagent (Bioteke Biotech, Beijing, China, RP1001). One microgram of total RNA was applied to synthesize cDNA using an All-in-One First-Strand SuperMix Kit according to the manufacturer’s instructions (Magen Biotechnology Co., Ltd., Guangzhou, China, MD80101). The 20-µl PCR system contained 1 µl of cDNA template, 0.5 µl of SYBR-GREEN I fluorochrome (Solarbio Life Sciences, Beijing, China, SR4110), 10 µl of 2×Fast Taq plus PCR Master Mix (Biosharp Technology Inc., BL1014), 0.5 µl of 10 µM forward primers, 0.5 µl of 10 µM reverse primers and 7.5 µl of sterile deionized H_2_O. The templates were amplified in 5 min at 95 °C, 40 cycles of 10 s at 95 °C, 10 s at 60 °C and 15 s at 72 °C, and last 90 s at 72 °C. Quantitative PCR data were analyzed by the 2(-Delta Delta C(T)) method [14] and GAPDH was used as the internal control gene to normalize the PCRs. Primers used in qPCR are shown in Supplementary Table S3.

To determine the mRNA stabilization of SMIT1, DLBCL-U2932 cells were treated with 2 µg/ml of transcription inhibitor Actinomycin D for 0, 2, 4, and 6 h, followed by qRCR analysis.

### RNA immunoprecipitation (RIP) assay

RIP assay was carried out using an EZ-Magna RIP Kit following the manufacturer’s instructions (Millipore Corp., Burlington, MA, USA, 17–701). Briefly, cells were lysed using RIP lysis buffer. To obtain magnetic bead-antibody complex, magnetic beads were incubated with antibodies against WTAP (16837-1-AP, Proteintech, China), YTHDF1 (ab220163, Abcam, Cambridge, USA) and control IgG (Millipore, Massachusetts, USA) at room temperature for 30 min. Then, the supernatant (10 µl) of cell lysate was immunoprecipitated with the magnetic bead-antibody complex overnight at 4 °C. Input was an unimmunoprecipitated control. The RNA-protein-bead complex was collected using a magnetic holder, eluted using RIP washing buffer and resuspended in protease K to purify RNA. The obtained RNA was used for RCR or qRCR analysis.

### Methylated RNA immunoprecipitation (MeRIP) assay

MeRIP was performed by using an m6A MeRIP Kit (BersinBio, Guangzhou, China, Bes5203-2). Briefly, total RNA from cells was extracted using TRIpure lysis buffer and fragmented by ultrasound. The anti-N6-methyladenosine (m6A) antibody or control IgG was conjugated to protein A/G magnetic beads. The fragmented RNAs were immunoprecipitated with antibody-protein A/G bead complex for 1 h at 4 °C. Input was an unimmunoprecipitated control. The RNA fragments bound to the protein A/G beads were extracted with elution buffer and purified with phenol-chloroform-isoamyl alcohol mixture, followed by qRCR analysis to detect the m6A-enriched content of the target RNA.

### Dual luciferase reporter assay

To verify the binding of WATP to SMIT1, a 200 bp fragment of SMIT1 3’UTR containing an m6A methylation site was synthesized and inserted into a pmirGLO luciferase reporter vector to construct a wild-type SMIT1 reporter plasmid. For mutation, the third base A within the m6A motif “GGACC” was replaced by a base C. The wild- or mutant-type SMIT1 reporter construct was co-transfected into HEK293T cells with Renilla luciferase vector and WATP-overexpressing plasmid or empty plasmid. After 48 h, firefly luciferase activity was detected using a Dual Luciferase Reporter Gene Assay Kit following the manufacturer’s instructions (Biosharp Technology Inc., BL555A) and normalized to that of renilla fluorescein.

### Western blot

Total protein was extracted from cells using RIPA buffer (Solarbio Life Sciences, R0010). The extracted protein samples (10–20 µg) were subjected to sodium dodecyl sulfate–polyacrylamide gel electrophoresis to separate proteins with different molecular weights. The proteins were then transferred to PVDF membranes (Millipore, IPVH00010) and treated with blocking solution (Solarbio Life Sciences, SW3010) for 1 h. The membranes were then incubated with primary antibodies at 4 °C overnight, followed by incubation of the horseradish peroxidase-conjugated secondary antibody at 37 °C for 45 min. Finally, the protein blots were visualized using chemiluminescence reagents (Solarbio Life Sciences, PE0010). The details of the antibodies used are listed in Table S4.

### Statistical analysis

Data are presented as mean ± SD. Statistical analyses were performed using GraphPad Prism (version 9.1). Data that passed any of the standard normality tests were analyzed with a two-tailed Student’s t test, one-way or two-way ANOVA followed by Tukey’s multiple comparison test. P-value < 0.05 was considered statistically significant.

## Results

### SMIT1 is upregulated in DLBCL tissues, indicating a worse prognosis

**Fig. 1:**
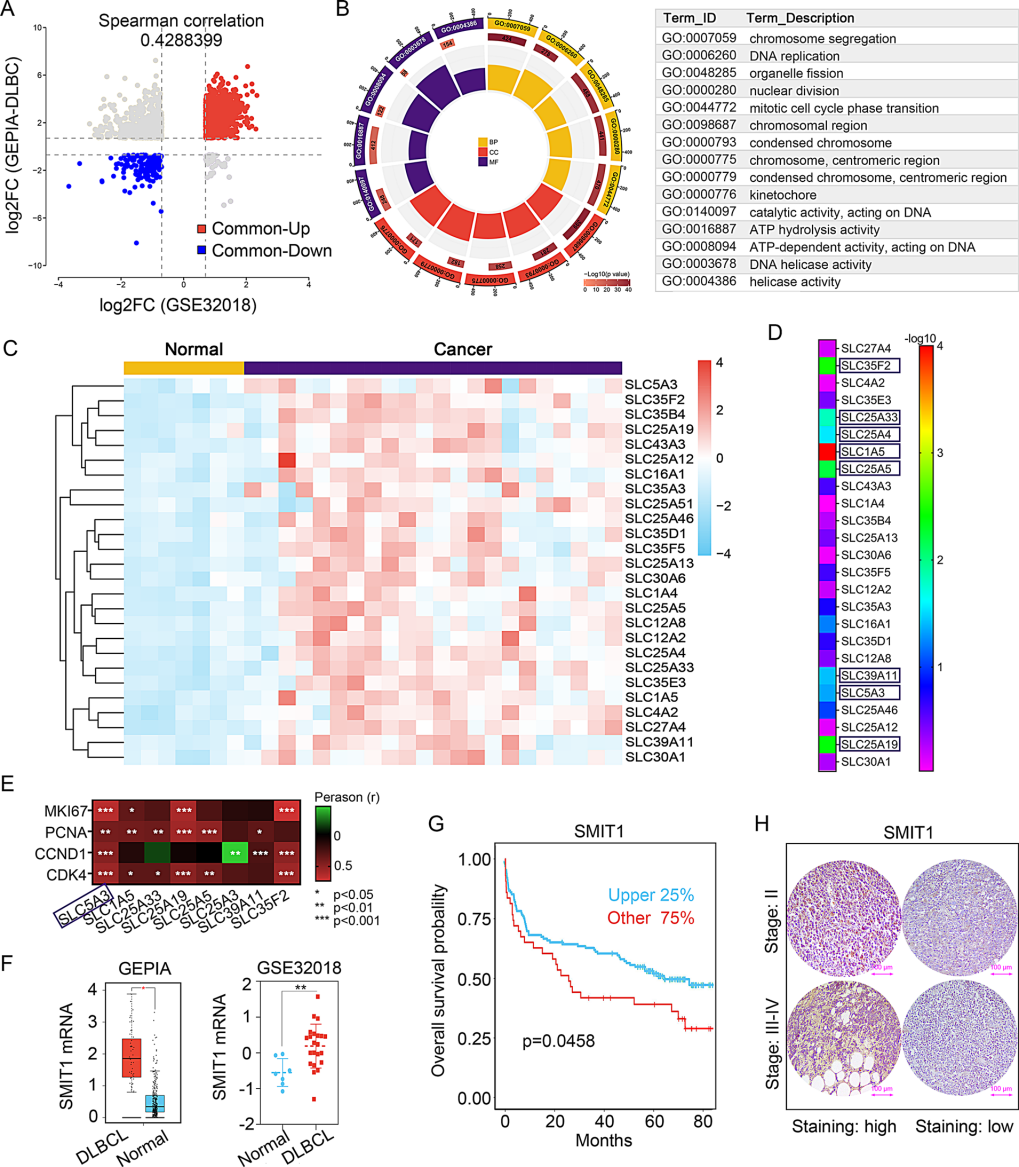
SMIT1 is upregulated in DLBCL tissues, indicating a worse prognosis. (**A**) Volcano plot showing the differently expressed genes in GEPIA-DLBCL and GSE32018 datasets with a screening criterion of >|1.5-fold change| and adj.*p* < 0.05. Red dot represents genes upregulated in GEPIA-DLBCL and GSE32018. Blue dot represents genes downregulated in GEPIA-DLBCL and GSE32018. (**B**) GO enrichment analysis of co-upregulated genes. (**C**) Heatmap showing the expression of co-upregulated SLCs in the GSE32018 database. (**D**) Survival heatmap showing the prognostic impacts of co-upregulated SLCs based on LOGpc database of DLBCL-GSE32918. The purple blocks represent significant (*p* < 0.05) unfavorable results. (**E**) Heatmap showing the correlation between 8 SLCs and survival-related markers in DLBCL based on GEPIA-DLBCL. expression of 8 SLCs in the GSE32018 database. (**F**) Expression of SMIT1 mRNA in GEPIA-DLBCL and GSE32018 datasets. (**G**) Overall survival curves showing the prognostic potential of SMIT1 for patients with DLBCL based on the LOGpc DLBCL-GSE32918 database. (**H**) Representative IHC staining images of SMIT1 in DLBCL tissues (200 ×)

Integrative analysis identified 1486 co-upregulated genes in GEPIA-DLBCL and GSE32018 datasets, showing >1.5-fold change and adj.*p* < 0.05 (Fig. 1A). GO enrichment analysis revealed these genes were primarily associated with cell cycle progression, DNA replication, and cellular division (Fig. 1B). Solute carrier (SLC) transporters, a superfamily of transmembrane proteins, play critical roles in maintaining cellular homeostasis through solute/ion translocation [15]. Focusing on co-upregulated SLC genes in DLBCL tissues, GSE32018 heatmap analysis identified 26 SLC family members with >1.5-fold upregulation and significantly differential expression (adj.*p* < 0.05; Fig. 1C). Using the DLBCL-GSE32918 cohort in the LOGpc database, 8 SLCs were associated with poor prognosis (Fig. 1D). Further analysis demonstrated that SLC5A3 (SMIT1) expression was significantly positively correlated with cell proliferation markers MKI67 (Ki-67), PCNA, CCND1 (cyclin D1), and CDK4 expression (Fig. 1E). As visualized in Fig. 1F and G, SLC5A3 upregulation was correlated with patients’ adverse outcomes. IHC analysis of 58 DLBCL specimens stratified patients into SMIT1-high and SMIT1-low subgroups, revealing SMIT1 overexpression in advanced Ann Arbor stage III-IV tumors (Fig. 1H; Tab. S1).

### SMIT1 facilitates DLBCL cell proliferation, cell cycle progression and inhibits cell apoptosis in vitro

**Fig. 2:**
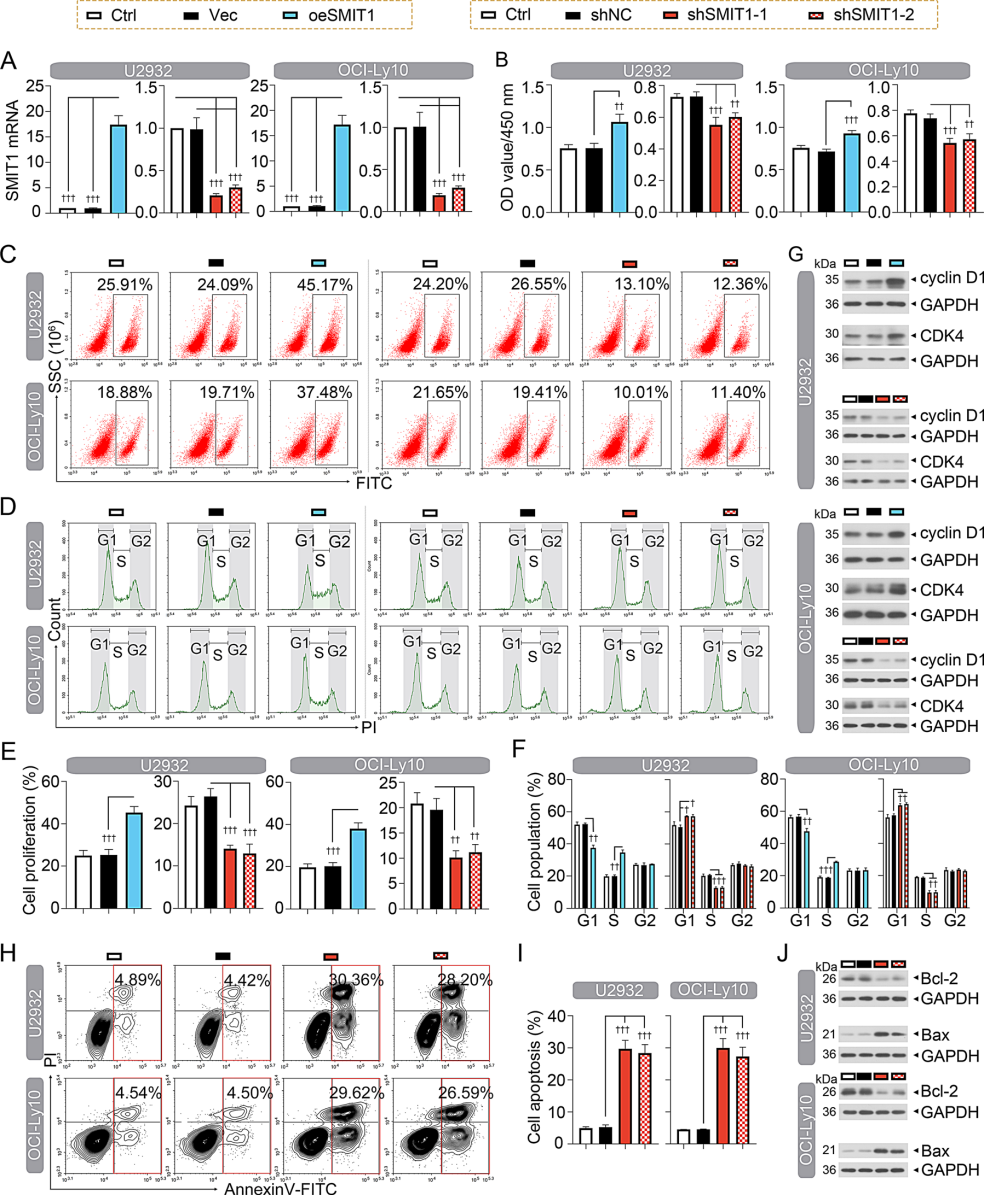
SMIT1 facilitates DLBCL cell proliferation, cell cycle progression and inhibits cell apoptosis in vitro. (**A**) qPCR assays showing the expression of SMIT1 mRNA in DLBCL-U2932 and DLBCL-OCI-Ly10 cells. (**B**) CCK8 cell viability assay was used for assessing DLBCL cell proliferation. (**C** and **E**) Measurement of DLBCL cell proliferation by Click-iT^®^ EdU flow cytometry assay. (**D** and **F**) Flow cytometry-based cell cycle detection and quantification in DLBCL cells. (**G**) Western blot assay showing the expression of cell cycle-related protein cyclin D1 and CDK4 in DLBCL cells. (**H** and **I**) Flow cytometry-based apoptosis detection and quantification in DLBCL cells. (**J**) Western blot assay showing the expression of apoptosis-related protein Bcl-2 and Bax in DLBCL cells. Data are presented as mean ± SD. ^†^ *p* < 0.05, ^††^ *p* < 0.01, and ^†††^ *p* < 0.001

To clarify the functional significance of SMIT1 in DLBCL, SMIT1 was highly expressed and knocked down in two DLBCL cell lines (U2932 and OCI-Ly10) by eukaryotic overexpression or interference systems (Fig. 2A). Results of CCK8 cell viability assay revealed that proliferative capacity of two DLBCL cells was enhanced after SMIT1 overexpression, yet inhibited after SMIT1 silencing (Fig. 2B). In addition, increased fluorescent intensity was observed during EdU incubation when SMIT1 was overexpressed (Fig. 2C and E). EdU-labelled proliferative cells were decreased when SMIT1 was knocked down (Fig. 2C and E). High SMIT1 expression also stimulated cell cycle progression, whereas low SMIT1 expression induced G1-S phase cell cycle arrest (Fig. 2D and F). Western blot analysis revealed that overexpression of SMIT1 upregulated the expression of cyclin D1 and its partner, CDK4, enabling cells to enter the S phase (Fig. 2G). Silencing of SMIT1 downregulated the expression of cyclin D1 and CDK4 (Fig. 2G). Flow cytometry-based apoptosis detection revealed that SMIT1 silencing increased the number of apoptotic cells (Fig. 2H and I). Moreover, SMIT1 silencing reduced the expression of anti-apoptotic protein Bcl-2 and enhanced the expression of pro-apoptotic protein Bax (Fig. 2J).

### SMIT1 accelerates DLBCL cell-derived tumor xenograft growth in vivo

**Fig. 3:**
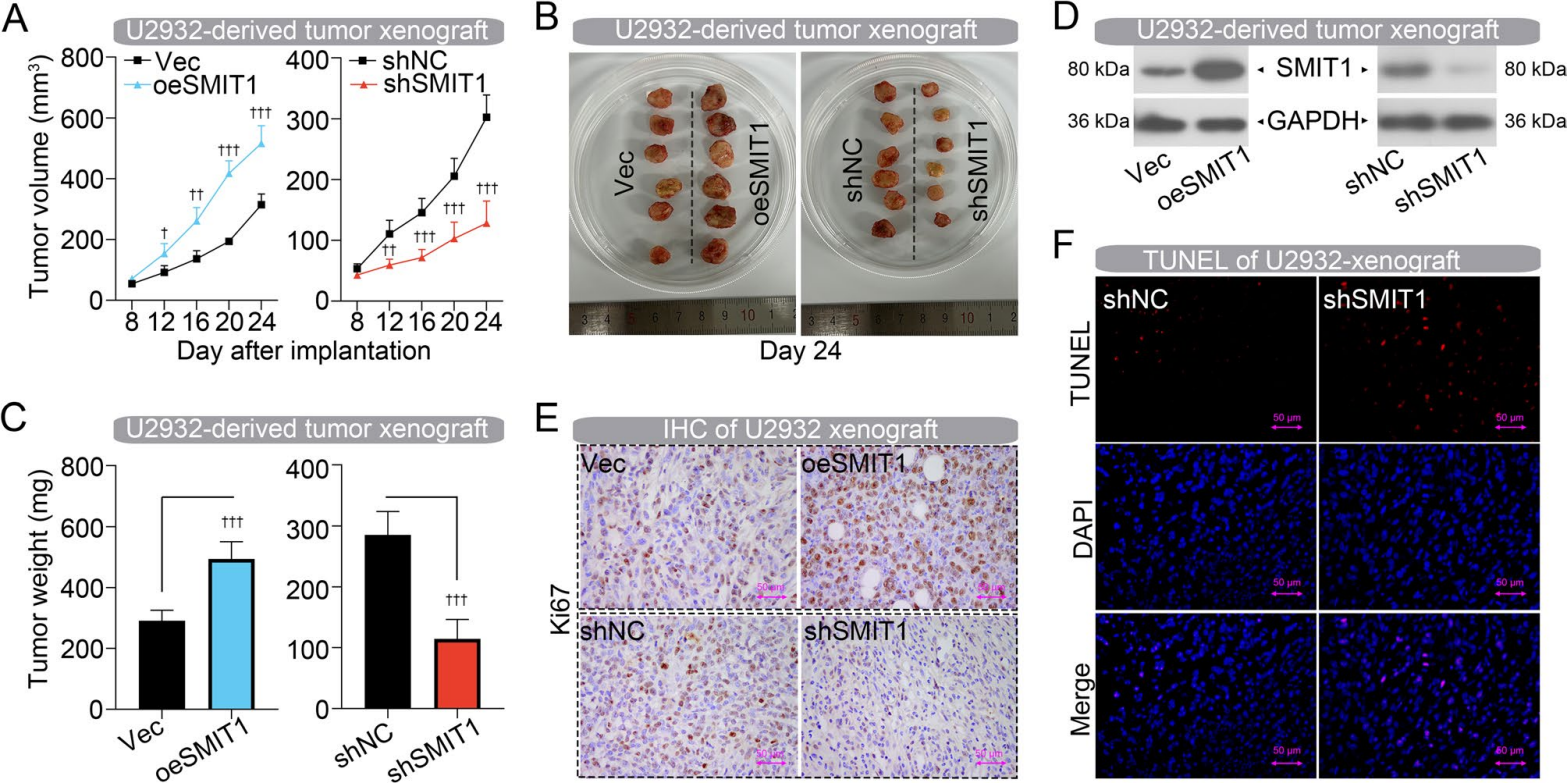
SMIT1 accelerates DLBCL cell-derived tumor xenograft growth in vivo. (**A**) Growth curves of tumor xenografts formed by U2932 cells bearing oeSMIT1 and shSMIT1. (**B**) Photographs of tumor xenografts on day 24 after implantation. (**C**) Tumor weight of xenografts on day 24 after implantation. (**D**) Western blot assay showing the expression of SMIT1 in tumors formed by U2932 cells. (**E**) IHC staining showing Ki67 expression in tumors formed by U2932 cells. (**F**) TUNEL staining in tumors formed by U2932 cells. Data are presented as mean ± SD. ^†^ *p* < 0.05, ^††^ *p* < 0.01, and ^†††^ *p* < 0.001

The tumorigenicity of SMIT1 was further evaluated in U2932 cell-derived tumor xenografts. SMIT1-overexpressed U2932 cells formed tumors at a much faster rate than the control cells (Fig. 3A). The volume and weight of tumors derived from SMIT1-overexpressed cells were significantly bigger than those derived from control cells on day 24 after implantation (Fig. 3B and C). Consistently, xenografts formed by U2932 cells bearing shSMIT1 exhibited a significantly slower growth rate (Fig. 3A) and lower tumor weight (Fig. 3B and C) than controls. Western blot assay verified expression of SMIT1 in U2932-derived tumor xenografts (Fig. 3D). Subsequently, histological evaluation suggested more Ki67-positive cells were present within the tumor mass formed by SMIT1-overexpressed U2932 cells, while fewer were present in SMIT1-silenced U2932 tumors (Fig. 3E). In addition, more TUNEL-positive cells in tumors derived from SMIT1-silenced U2932 cells confirmed its pro-apoptotic capability (Fig. 3F).

### SMIT1 induces AKT signaling activation

**Fig. 4:**
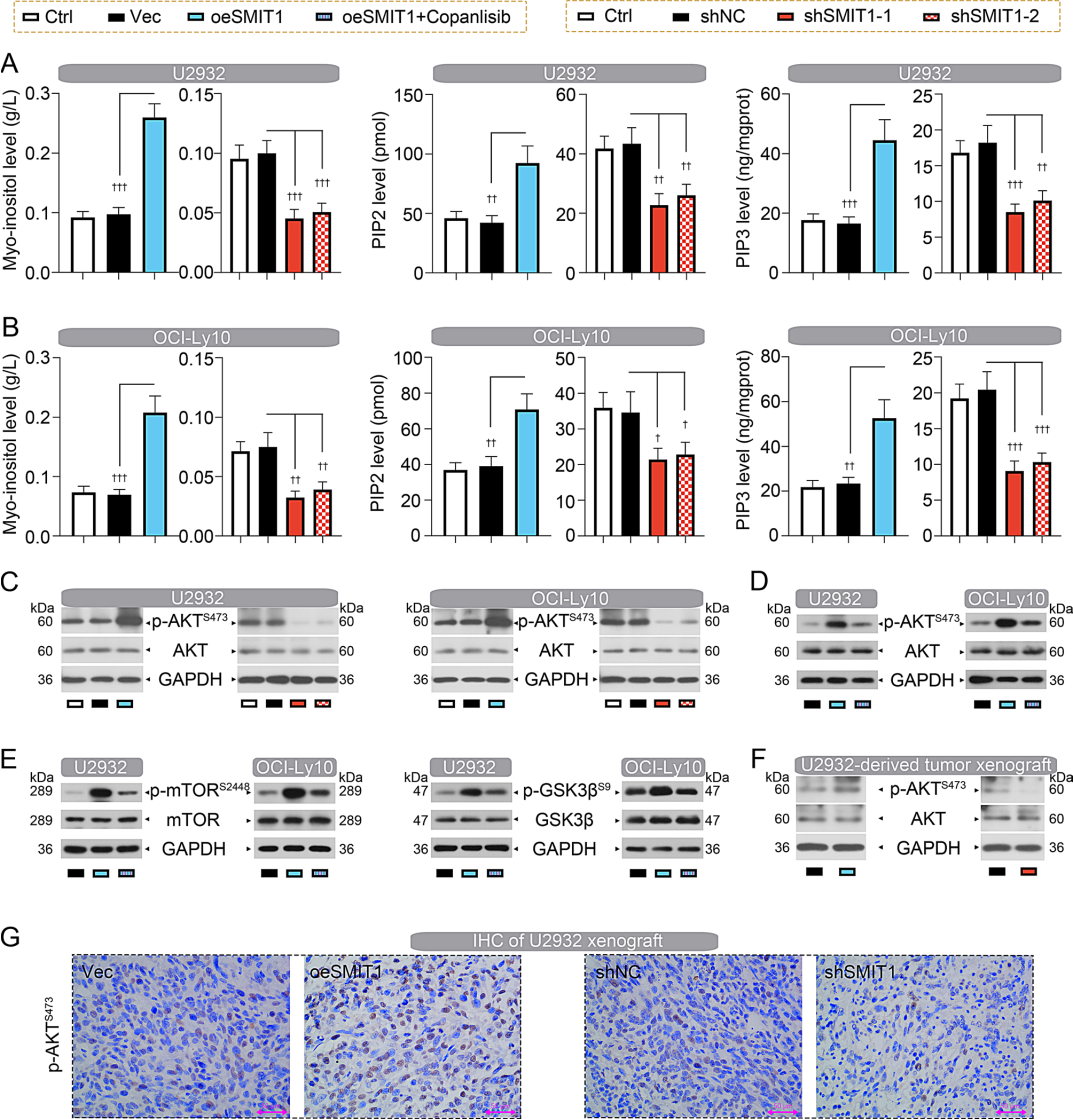
SMIT1 induces AKT signaling activation. (**A**) Levels of myo-inositol, PIP2 and PIP3 in U2932 cells. (**B**) Levels of myo-inositol, PIP2 and PIP3 in OCI-Ly10 cells. (**C**) Western blot assay showing the expression levels of phospho-AKT (Ser473) and total AKT in U2932 and OCI-Ly10 cells. (**D**) Western blot assay showing the expression levels of phospho-AKT (Ser473) in U2932 and OCI-Ly10 cells in the presence and absence of PI3K inhibitor Copanlisib. (**E**) Western blot assay showing the expression levels of phospho-mTOR (Ser2448) and phospho-GSK3β (Ser9) in U2932 and OCI-Ly10 cells in the presence and absence of PI3K inhibitor Copanlisib. (**F**) Western blot assay showing the expression levels of phospho-AKT (Ser473) and total AKT in U2932 cell-derived tumor xenograft. (**G**) IHC staining showing phospho-AKT (Ser473) expression in tumors formed by U2932 cells. Data are presented as mean ± SD. ^†^ *p* < 0.05, ^††^ *p* < 0.01, and ^†††^ *p* < 0.001

Overexpression of SMIT1 promoted intracellular accumulation of myo-inositol, increased phosphoinositide levels (PIP2 and PIP3), while knockdown of SMIT1 yielded the opposite results in DLBCL-U2932 cells (Fig. 4A) and DLBCL-OCI-Ly10 cells (Fig. 4B). Generation of phosphatidylinositol PIP2 and PIP3 can activate AKT signaling, which in turn plays key roles in carcinogenesis [5]. Here, we observed that phospho-AKT (Ser473) level was increased following SMIT1 overexpression, while decreased following SMIT1 silencing in U2932 and OCI-Ly10 cells (Fig. 4C). To further confirm whether SMIT1-mediated AKT activation was dependent on the PI3K pathway, we treated SMIT1-overexpressing U2932 and OCI-Ly10 cells with the PI3K inhibitor Copanlisib. This treatment notably decreased the phosphorylation levels of AKT and its downstream effectors (mTOR, GSK3β; Fig. 4D, E). U2932-derived xenografts showed consistent changes in AKT phosphorylation (Fig. 4F, G).

### m6A modification of SMIT1 enhanced its stability

**Fig. 5:**
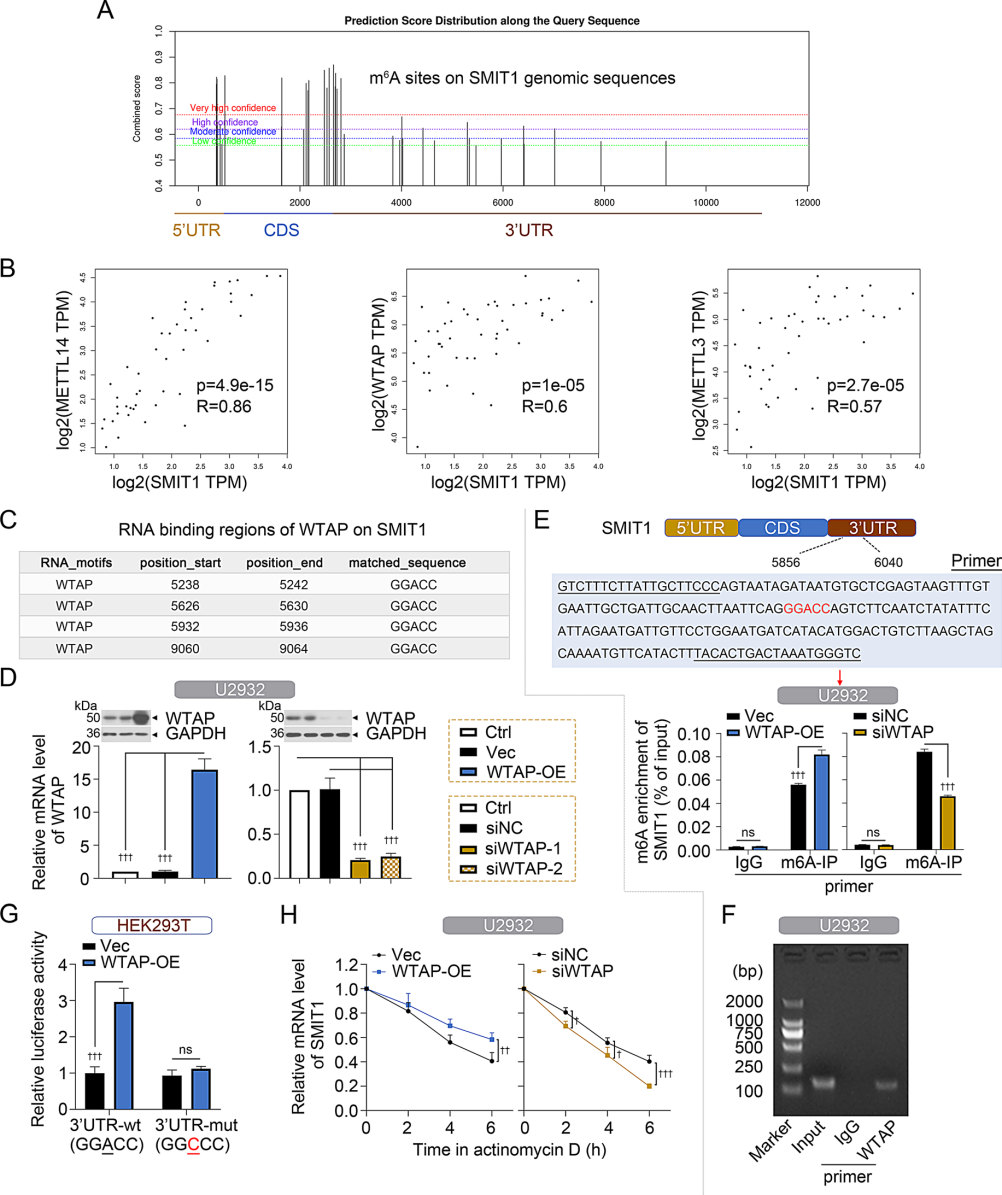
WTAP-mediated m6A modification of SMIT1 enhances its stability. (**A**) The m6A modification site of SMIT1 mRNA predicted by the Sequence-Based Predictor of RNA Adenosine Methylation Sites (SRAMP) online tool. (**B**) Correlation analysis between SMIT1 and METTL3/METTL14/WTAP mRNA expression in DLBCL from GEPIA2.0 database. (**C**) catRAPID fragment-based prediction analysis of interaction between SMIT1 and WTAP. (**D**) qPCR and western blot analyses showing WTAP mRNA and protein levels in DLBCL cells upon WTAP overexpression (WTAP-OE, vector) and WTAP knockdown (si-WTAP-1, si-WTAP-2, siNC). (**E**) MeRIP-qPCR analysis showing m6A enrichment of SMIT1 mRNA in U2932 cells upon WTAP overexpression and knockdown. (**F**) RIP-PCR analysis showing an interaction between SMIT1 and WTAP in DLBCL U2932 cells. (**G**) Dual-luciferase reporter gene assay showing SMIT1 mRNA in HEK293T cells transfected the wild-type or mutant SMIT1 reporter vectors. (**H**) qPCR and actinomycin D assays showing the expression and stability of SMIT1 mRNA in DLBCL U2932 cells upon WTAP overexpression (WTAP-OE, vector) and WTAP knockdown (si-WTAP-1, siNC). Data are presented as mean ± SD. ^†^ *p* < 0.05, ^††^ *p* < 0.01, and ^†††^ *p* < 0.001. ns, no significance

We explored the potential mechanism of SMIT1 upregulation in DLBCL. Previous research has shown that m6A modification plays a pivotal role in RNA translation, splicing, and degradation [16]. SRAMP prediction website (http://www.cuilab.cn/sramp/) displayed high-confidence m6A modification sites of SMIT1 (Fig. 5A). We analyzed the expression correlation between SMIT1 and three essential components of the human m6A methyltransferase complex (METTL3, METTL14, and WTAP) in GEPIA-DLBCL [17]. Result revealed that SMIT1 mRNA level was positively correlated with three key subcomplexes (correlation coefficient *R* = 0.89/0.6/0.57 for METTL3/METTL14/WTAP, respectively, all *p* < 0.001) (Fig. 5B). Since WATP was known as an oncogene in DLBCL [18], we chose WTAP for the follow-up study. catRAPID fragment-based prediction analysis of interaction between SMIT1 and WTAP revealed four high-confidence m6A motifs (GGACC) on 3’UTR of SMIT1 mRNA (Fig. 5C). To determine whether WTAP regulates m6A modification of SMIT1, we firstly constructed WTAP-overexpressing plasmids and two RNA-interference plasmids targeting human WTAP mRNA to manipulate gene expression (Fig. 5D). Subsequently, MeRIP-qPCR assay was performed, in which primers were designed to amplify the fragment near 5932–5936 of m6A position (Fig. 5E). A basal m6A modification was detectable on SMIT1 mRNA. Such m6A modification was enhanced after exogenous WTAP overexpression, yet inhibited after WTAP silencing (Fig. 5E). RIP-PCR assay showed that WTAP bound to SMIT1 mRNA (Fig. 5F). The pmirGLO Dual-luciferase expression vector was designed by the insertion of SMIT1 sequence containing the wild- or mutant-type m6A site, and transfected into HEK293T cells with WTAP-overexpressing plasmids, to study the influence of WTAP-mediated m6A modulation on SMIT1 mRNA stability. As shown in Fig. 5G, luciferase activity was significantly increased in HEK293T cells cotransfected with WTAP-overexpressing plasmids and wild-type SMIT1 reporter vectors, while there was no difference in the mutant type. qPCR and actinomycin D assays showed that the expression and stability of SMIT1 were enhanced after WTAP overexpression, yet reduced after WTAP silencing (Fig. 5H).

### The reader protein YTHDF1 mediates m6A modification and stability of WTAP on SMIT1 mRNA

**Fig. 6:**
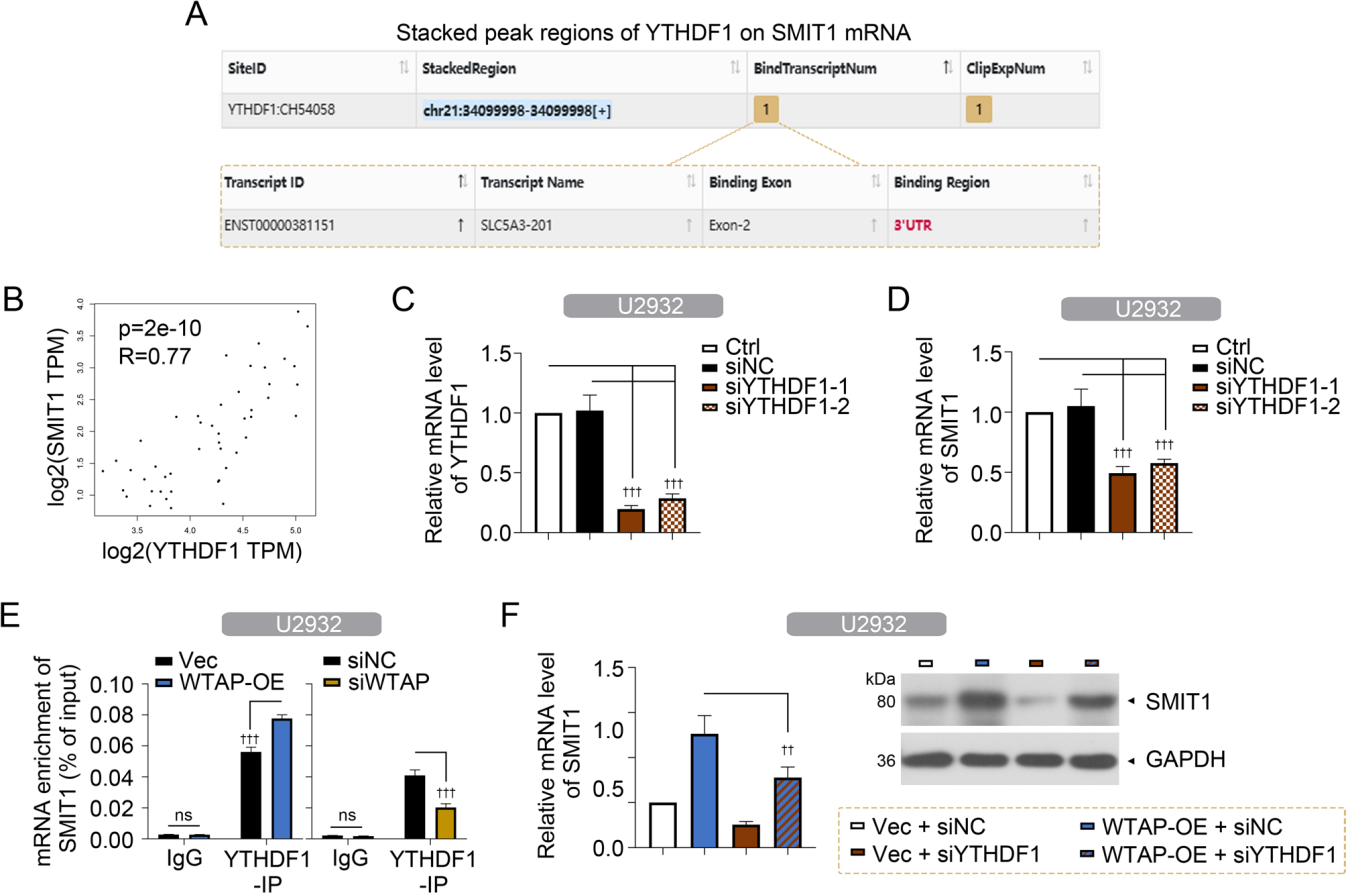
The reader protein YTHDF1 mediates m6A modification and stability of WTAP on SMIT1 mRNA. (**A**) The binding region of SMIT1 mRNA with YTHDF1 protein predicted by Encyclopedia of RNA Interactomes (ENCORI/starBase) website. (**B**) Correlation between SMIT1 and YTHDF1 mRNA expression in DLBCL from GEPIA2.0 database. (**C** and **D**) qPCR assays showing the expression of YTHDF1 (**C**) and SMIT1 (**D**) mRNA in DLBCL U2932 cells. (**E**) RIP-qPCR analysis showing an interaction between SMIT1 and YTHDF1 in U2932 cells. (**F**) qPCR and western blot assays showing the expression of SMIT1 mRNA and protein in U2932 cells. Data are presented as mean ± SD. ^†^ *p* < 0.05, ^††^ *p* < 0.01, and ^†††^ *p* < 0.001. ns, no significance

RNAs with m6A modification can be recognized by m6A ‘readers’ such as YTHDF1, 2, and 3. ENCORI website showed that YTHDF1, 2, and 3 bound to SMIT1 mRNA (Fig. 6A and Fig. S1A). Data form GEPIA-DLBCL revealed that SMIT1 mRNA level was positively correlated with YTHDF1 mRNA level in DLBCL tissues (Fig. 6B). Next, we detected the effects of YTHDF1, 2, and 3 on SMIT1 mRNA expression after knocking each of them down in DLBCL cells (Fig. 6C and Fig. S1B-C). Results showed that the silencing of YTHDF1, but not YTHDF2 or 3, downregulated SMIT1 mRNA expression (Fig. 6D and Fig. S1B-C). RIP-qPCR assays using anti-YTHDF1 antibody suggested that YTHDF1 bound to SMIT1 mRNA (Fig. 6E). Moreover, WTAP overexpression enhanced the interaction of YTHDF1 with SMIT1, whereas silencing of WTAP inhibited this process (Fig. 6F). Following qPCR and western blot assays confirmed that knockdown of “readers” YTHDF1 inhibited upregulation of SMIT1 expression caused by WTAP overexpression (Fig. 6G). Our findings suggested that SMIT1 mRNA stability was associated with WTAP/YTHDF1-mediated m6A modification.

### Silencing of SMIT1 inhibits DLBCL progression by disturbing AKT activity

**Fig. 7:**
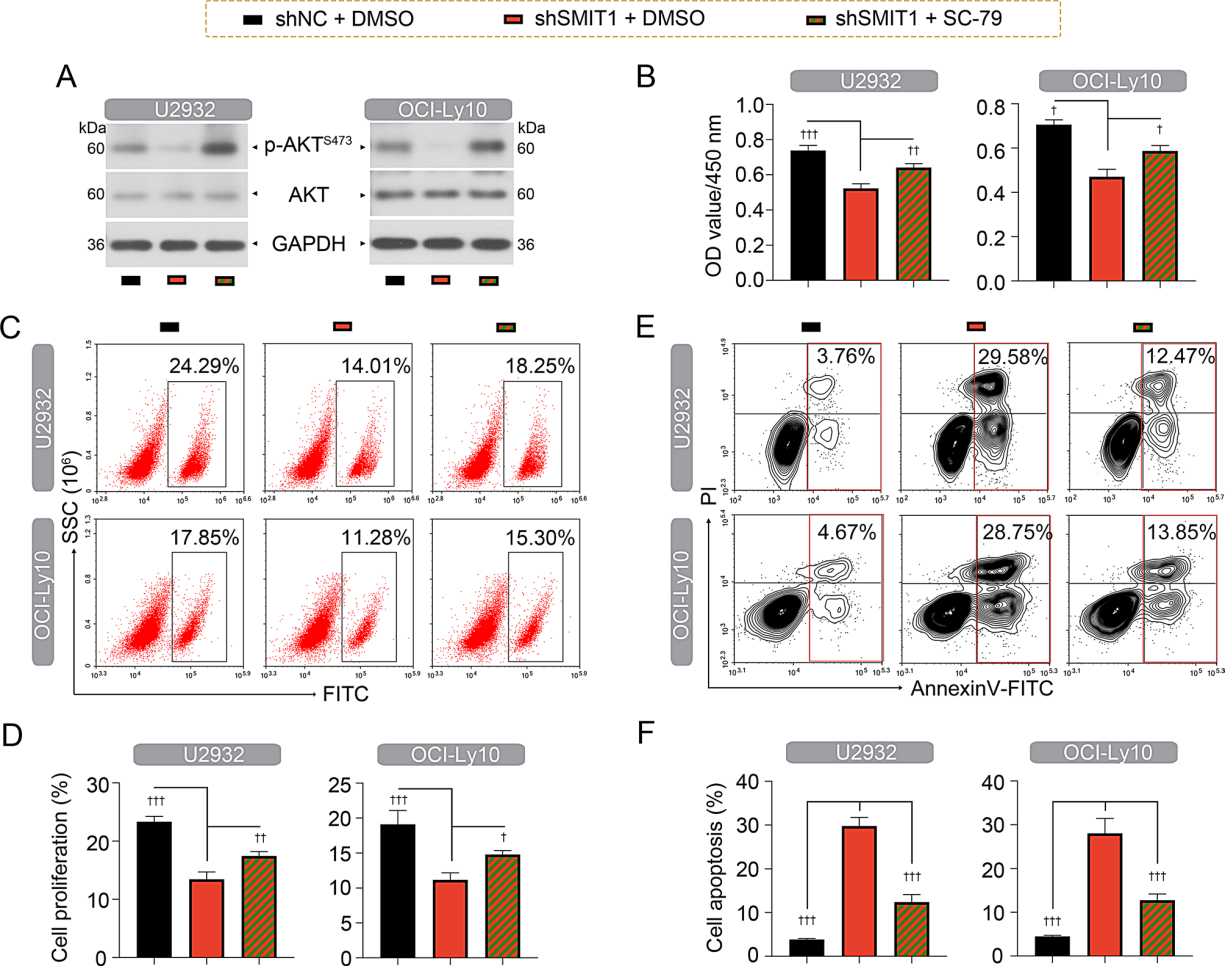
SMIT1 facilitates DLBCL progression by activating AKT signaling. (**A**) Western blot assay showing the expression levels of phospho-AKT (Ser473) and total AKT in U2932 cells. (**B**) CCK8 cell viability assay at 24 h was used for assessing cell proliferation. (**C** and **D**) Measurement of proliferative cells by Click-iT^®^ EdU flow cytometry assay. (**E** and **F**) Flow cytometry-based apoptosis detection and quantification in U2932 cells. Data are presented as mean ± SD. ^†^ *p* < 0.05, ^††^ *p* < 0.01, and ^†††^ *p* < 0.001

Further experiments were performed to uncover the underlying mechanism of SMIT1 in DLBCL progression using the AKT agonist SC-79, which allows AKT to be phosphorylated by upstream kinases in the cytoplasm. Administration of SC-79 reversed SMIT1 silencing-caused downregulation of phospho-AKT (Ser473) expression in U2932 and OCI-Ly cells (Fig. 7A). Functional experiments showed that SC-79 inhibited anti-proliferative (Fig. 7B-D) and pro-apoptotic (Fig. 7E and F) roles of SMIT1 silencing in U2932 and OCI-Ly cells.

## Discussion

DLBCL is a considerably heterogeneous lymphoid malignancy characterized by a wide range of morphologies and molecular attributes. Hence, elucidating the molecular mechanism of DLBCL tumorigenesis and progression holds important value in basic theory and clinical applications. Recently, the molecular mechanisms of the SLC family in tumorigenesis have been uncovered in diverse tumor types. In this study, our objective was to identify SMIT1, which might serve as an independent biomarker for the tumorigenesis and progression of DLBCL. Our findings reveal its roles in DLBCL cell proliferation, cell cycle progression and apoptosis.

It is known that the main function of the SLC family is to facilitate the transport of various substrates across biological membranes. We have observed that certain members of the SLC subfamilies exhibit high mRNA levels in DLBCL. Specifically, these include members of the nucleotide sugar transporter family (SLC35F2, SLC39A11), members of the mitochondrial transporter family (SLC25A3, SLC25A5, SLC25A19, SLC25A33), members of the amino acid transporter family (SLC1A5), and sodium-coupled inositol transporter proteins (SMIT1). These molecules have been designated as potential therapeutic targets for various malignancies [13, 19–25]. The following survival analysis reveals that elevated expression of SMIT1 is associated with the unfavorable survival prognosis of DLBCL. Moreover, SMIT1 displays the most significant positive correlation with cell survival-related markers such as Ki67, PCNA, cyclin D1 and CDK4. Therefore, we hypothesized that SMIT1 might contribute to the progression of DLBCL.

m6A modification of gene transcripts plays critical roles in DLBCL [26]. m6A modification was controlled by the “writers” m6A methyltransferases, “erasers” m6A demethylases, and “readers” binding proteins to regulate RNA splicing, nuclear exportation, translation, and degradation [16]. The “writers” WTAP was upregulated in DLBCL tissues and promoted the malignant phenotypes of DLBCL cells due to its m6A demethylase activity [18]. We found that WTAP and SMIT1 exhibited a significantly positive correlation in mRNA expression from the GEPIA database. Subsequent experiments suggested that SMIT1 was the critical target gene of WTAP. Inhibition of WTAP reduced the m6A level of SMIT1, which led to the downregulation of SMIT1 at the RNA level. Previous studies suggest that mRNA transcripts with m6A modifications tend to be stable or decaying, largely due to regulation by m6A readers such as YTHDFs. Here, silencing of YTHDF1, but not YTHDF2 or 3, reduced the expression of SMIT1 in DLBCL cells. Moreover, the binding of YTHDF1 to m6A-modified SMIT1 has been confirmed in DLBCL cells. Our study indicates that the mRNA stability of SMIT1 is controlled by WTAP/YTHDF1. Whether other “writers”, “erasers” or “readers” participate in the regulation of SMIT1 needs to be further investigated.

SMIT1 is responsible for transporting inositol from the cell membrane into the cell and thereby promoting tumor growth [13]. It is documented that myo-inositol induces activation of Akt signaling [6]. The AKT signaling cascade is triggered in DLBCL [7, 8]. In this process, the phosphorylated PI3K converts PIP2 to PIP3, which activates AKT. AKT decreases the expression of pro-apoptotic proteins, such as BCL-2-antagonist of cell death (BAD), BCL-2-like protein 11 (BIM), procaspase-9, and forkhead box protein O1 (FoxO1), thereby blocking apoptosis. During cell cycle progression, AKT activation leads to the phosphorylation of glycogen synthase kinase 3β (GSK3β), which in turn prevents the degradation of cyclin D1 [27, 28]. Although PI3K is the primary activator of AKT signaling, several stimuli and kinases initiate AKT signaling to drive cellular survival, proliferation, invasion, and apoptosis [29]. It has been suggested that SMIT1 causes AKT activation in tumor cells [11, 12]. In addition, SMIT1 silencing induces cleavages of caspase-3, poly (ADP-ribose) polymerase (PARP) and caspase-9, while provoking mitochondrial depolarization in NSCLC cells [11]. SMIT1 silencing also induces G1-S arrest of NSCLC cells [11]. In the present study, we confirm that knockdown of SMIT1 restrains cell proliferation, initiates caspase-mediated apoptosis activation and cell cycle arrest in vitro, and suppresses tumor growth in vivo by disrupting phosphatidylinositol homeostasis and the ensuing AKT activity.

The tumor suppressor PTEN can dephosphorylate PIP3 back to PIP2 and prevent AKT recruitment [30]. The ABC-subtype DLBCL has a relatively high incidence rate in clinical practice. Here, we choose a PTEN-deficient ABC-DLBCL cell line (U2932) and a PTEN-positive ABC-DLBCL cell line (OCI-Ly10). Results revealed that knockdown of SMIT1 induced proliferation inhibition and apoptosis in U2932 and OCI-Ly10 cells via provoking the activation of AKT signaling, suggesting that SMIT1 may serve as a novel therapeutic target for ABC-type of DLBCL, regardless of PTEN expression status. Whether SMIT1 can act as a universal target across all DLBCL subtypes (e.g., GCB type) requires further investigation in future studies.

This study has several limitations. First, the prognostic significance of SMIT1 in DLBCL requires further validation with an expanded clinical sample cohort. For instance, collecting fresh frozen specimens to validate SMIT1 expression at both the mRNA and protein levels, thereby better exploring its potential clinical value; and increasing the sample size to incorporate additional key clinical parameters (e.g., IPI score, MYC/Bcl-2 double expression, Ki-67 index) for multivariate analyses, which would enhance the robustness of conclusions regarding SMIT1’s clinical relevance. Second, to further clarify the regulatory role of SMIT1 in the AKT pathway, in vivo experiments—such as a combined treatment group (e.g., AKT agonist plus SMIT1 knockdown) in the tumor xenograft model—would help verify whether SMIT1 exerts its effects in an AKT-dependent manner. We are committed to addressing them in our future research to improve the clinical translational potential of our findings.

## Conclusions

We demonstrate that SMIT1 expression is significantly upregulated in DLBCL tissues, and that its RNA expression and stabilization are regulated by WTAP/YTHDF1-mediated m6A modification. Knockdown of SMIT1 exhibits anti-proliferative and pro-apoptotic effects at least by disrupting phosphatidylinositol homeostasis and the ensuing AKT activity.

## Supplementary Information

Below is the link to the electronic supplementary material.

Supplementary Material 1

Supplementary Material 2

Supplementary Material 3

## Data Availability

The data that support the findings of this study are available from the corresponding author on reasonable request.
