# Supplementary figures and images for: Sodium-myoinositol cotransporter-1 downstream of m6A methyltransferase WTAP exerts a potential carcinogenicity in diffuse large B-cell lymphoma progression
Source: J Transl Med. 2025 Nov 18;23:1310. doi: 10.1186/s12967-025-07303-7 (PMC12625369; doi:10.1186/s12967-025-07303-7)

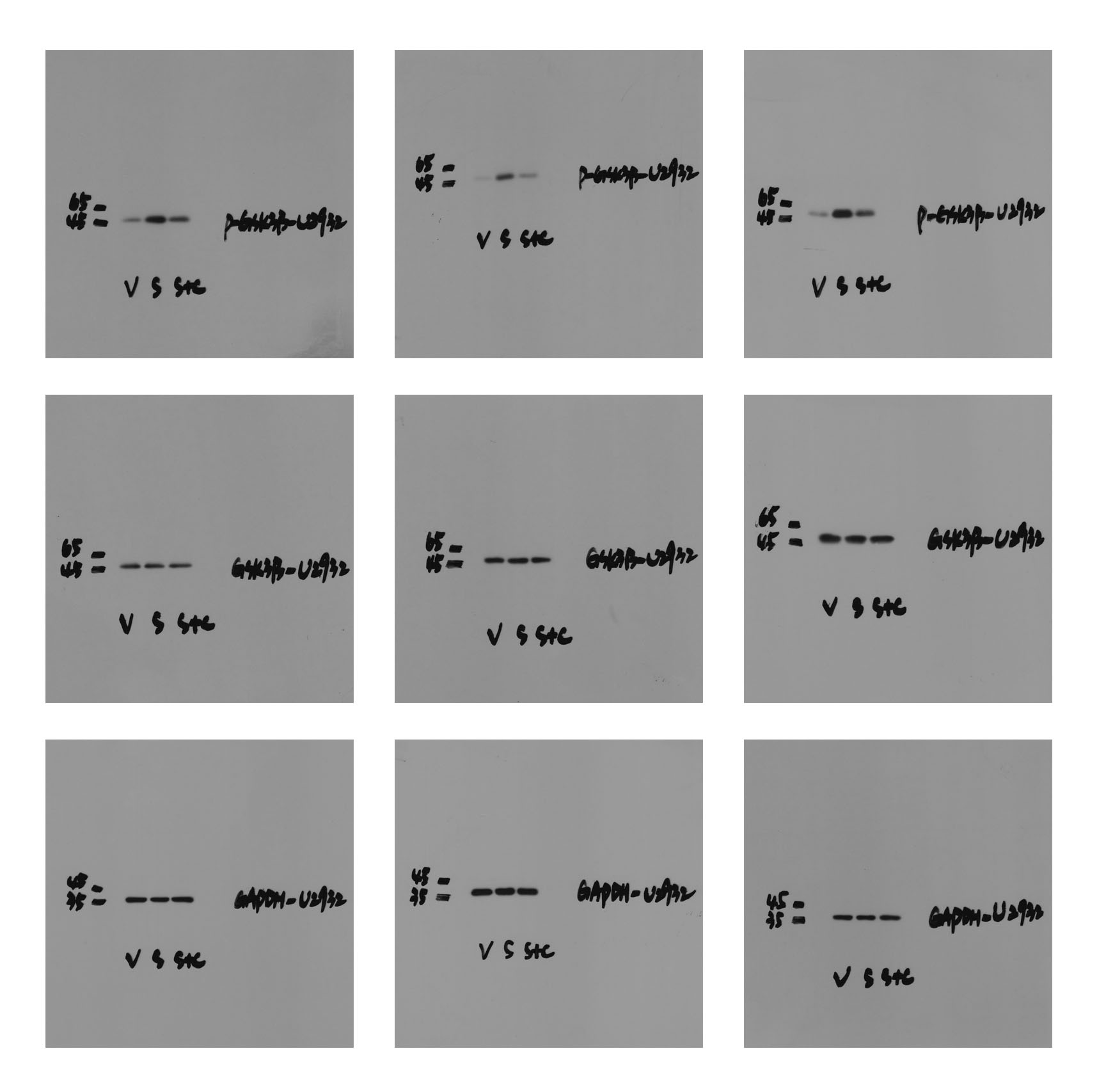

Supplement: Supplementary file 1 — Supplementary Material 1 [file 12967_2025_7303_MOESM1_ESM.jpg]

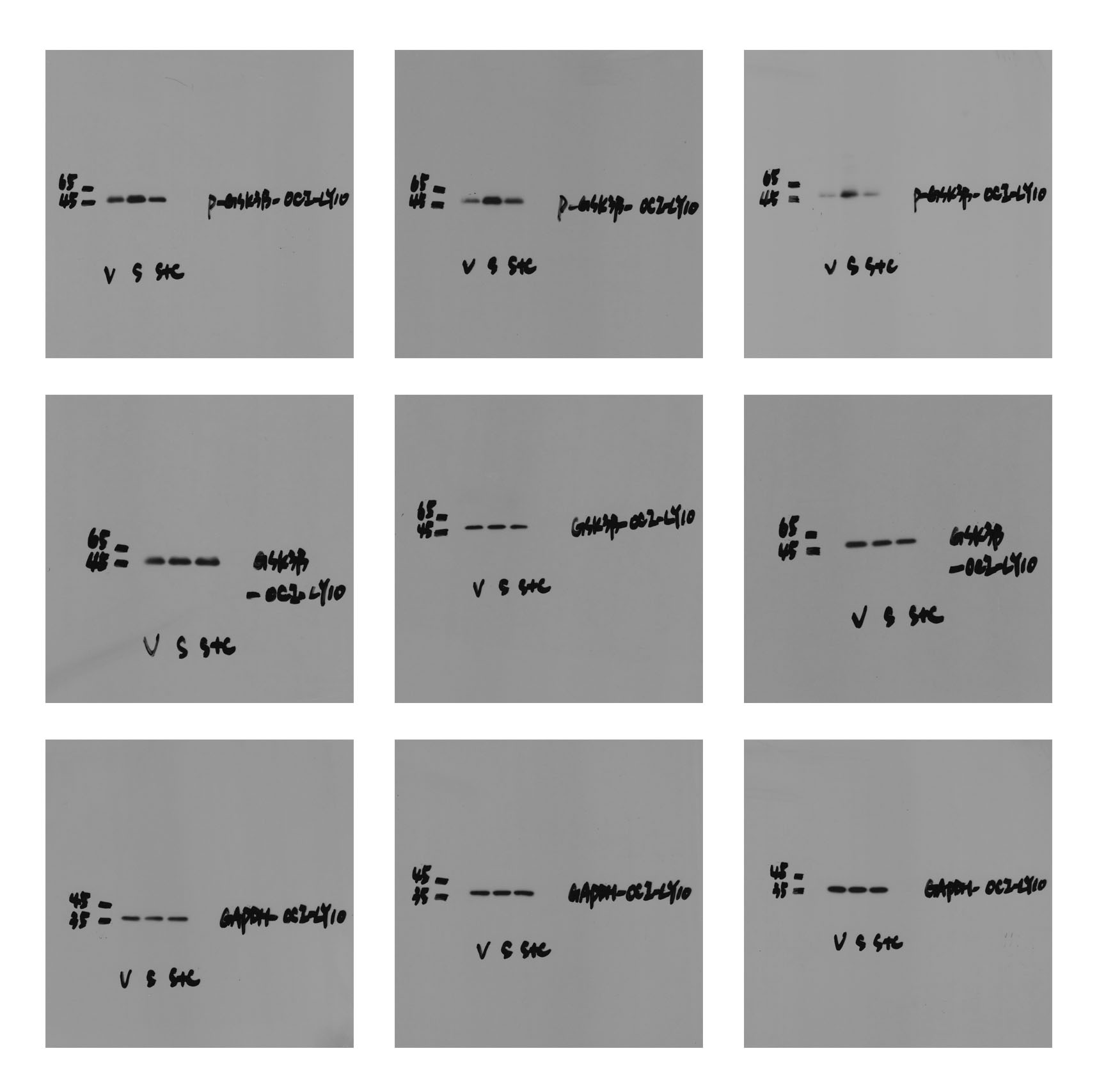

Supplement: Supplementary file 2 — Supplementary Material 2 [file 12967_2025_7303_MOESM2_ESM.jpg]
