# Supplementary material for: Sodium-myoinositol cotransporter-1 downstream of m6A methyltransferase WTAP exerts a potential carcinogenicity in diffuse large B-cell lymphoma progression
Source: J Transl Med. 2025 Nov 18;23:1310. doi: 10.1186/s12967-025-07303-7 (PMC12625369; doi:10.1186/s12967-025-07303-7)
Supplement: Supplementary file 3 — Supplementary Material 3 [file 12967_2025_7303_MOESM3_ESM.docx]

**Supplementary information**

Xinyang Li, Xiaowei He, Ying Sun, Wei Yang*

*Department of Hematology,* *Shengjing Hospital of China Medical University, Shenyang, China.*

*Correspondence

Wei Yang. Department of Hematology, Shengjing Hospital of China Medical University, 36# Sanhao Street, Shenyang, China. Tel: (+86)-24-96615-24115. Email: [sjyangw1012@163.com](mailto:sjyangw1012@163.com)

**
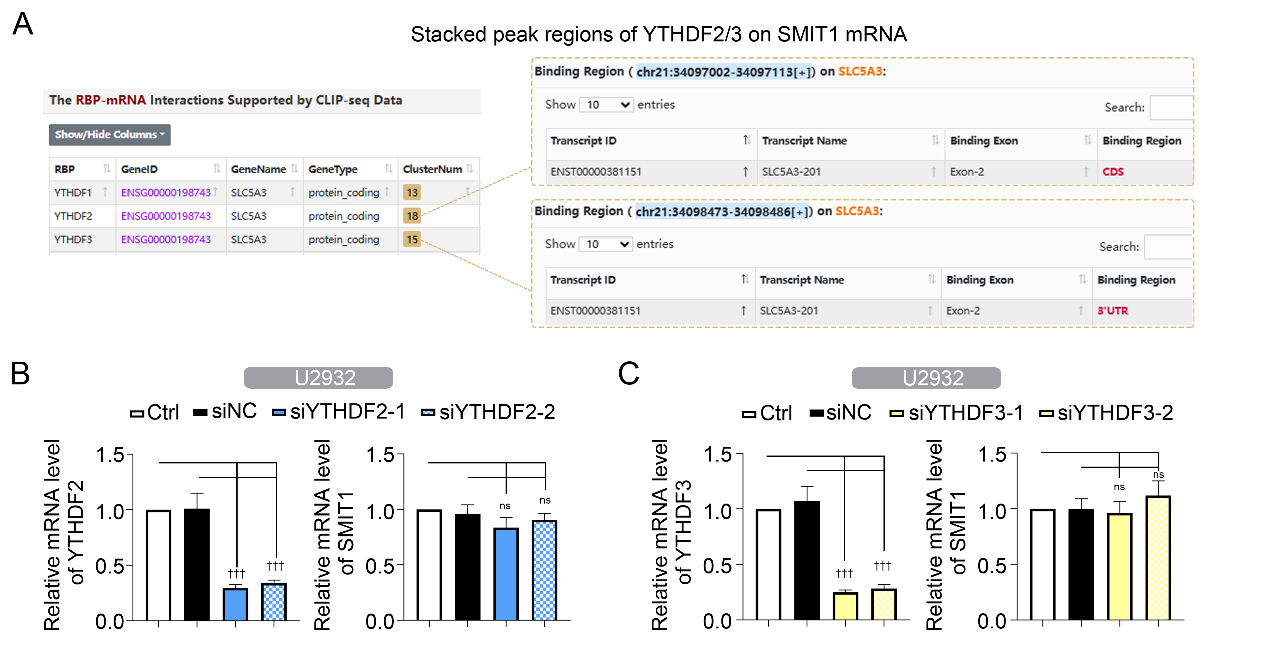
**

**Figure S1. Related to Figure 3. YTHDF2 and YTHDF3 do not affect expression of SMIT1 mRNA.**

(A) The binding region of SMIT1 mRNA with YTHDF2 and YTHDF3 protein predicted by ENCORI website. (B) qPCR assay showing the expression of YTHDF2 and SMIT1 mRNA in U2932 cells upon YTHDF2 silencing. (C) qPCR assay showing the expression of YTHDF3 and SMIT1 mRNA in U2932 cells upon YTHDF3 silencing. ^†^ *p* < 0.05, ^††^ *p* < 0.01, and ^†††^ *p* < 0.001. ns, no signiﬁcance.

**Table S1. The correlation between SMIT1 and clinicopathological factors in patients with DLBCL.**

| **Parameters** | **n** | **SMIT1 expression** | | **P value** |
| --- | --- | --- | --- | --- |
|  |  | **Low(n)** | **High(n)** |  |
| **Age** |  |  |  | 0.2124 |
| <60 | 32 | 15 | 17 |  |
| ≥60 | 26 | 8 | 18 |  |
| **Ann Arbor Stage** |  |  |  | **0.0146** |
| Ⅰ, Ⅱ | 24 | 14 | 10 |  |
| Ⅲ, Ⅳ | 34 | 9 | 25 |  |
| **Tumor diameter** |  |  |  | 0.6301 |
| <5 cm | 28 | 12 | 16 |  |
| ≥5 cm | 30 | 11 | 19 |  |
| **LDH** |  |  |  | 0.1060 |
| Normal | 41 | 19 | 22 |  |
| Elevated | 17 | 4 | 13 |  |
| **B symptoms** |  |  |  | 0.5610 |
| Absent | 43 | 18 | 25 |  |
| Present | 15 | 5 | 10 |  |
| **IPI score** |  |  |  | 0.1060 |
| 0-2 | 41 | 19 | 22 |  |
| 3-5 | 17 | 4 | 13 |  |
| **ECOG score** |  |  |  | 0.1058 |
| 0-1 | 47 | 21 | 26 |  |
| 2-3 | 11 | 2 | 9 |  |
| **Extranodal sites** |  |  |  | 0.3510 |
| 0-1 | 47 | 20 | 27 |  |
| 2-4 | 11 | 3 | 8 |  |

DLBCL, diffuse large B-cell lymphoma; IPI, International Prognostic Index; ECOG, Eastern Cooperative Oncology Group performance status; LDH, lactic dehydrogenase. The bold values showed statistically significant.

**Table S2. The sequences for shRNA or siRNA.**

| **siRNA/shRNA** | | **Sequence (5’-3’)** |
| --- | --- | --- |
| siWTAP-1 | Sense | GCAAGAGUGUACUACUCAA |
|  | Antisense | UUGAGUAGUACACUCUUGC |
| siWTAP-2 | Sense | GCGAAGUGUCGAAUGCUUA |
|  | Antisense | UAAGCAUUCGACACUUCGC |
| siYTHDF1#1 | Sense | ACAAGAUAAUAAAGUACAA |
|  | Antisense | UUGUACUUUAUUAUCUUGU |
| siYTHDF1#2 | Sense | CCUCCACCCAUAAAGCAUA |
|  | Antisense | UAUGCUUUAUGGGUGGAGG |
| siYTHDF2#1 | Sense | GCACAGAAGUUGCAAGCAA |
|  | Antisense | UUGCUUGCAACUUCUGUGC |
| siYTHDF2#2 | Sense | GCCCAAUAAUGCAUAUACU |
|  | Antisense | AGUAUAUGCAUUAUUGGGC |
| siYTHDF3#1 | Sense | GGUAUGACUAGCAUUGCAA |
|  | Antisense | UUGCAAUGCUAGUCAUACC |
| siYTHDF3#2 | Sense | GACUAGCAUUGCAACCAAU |
|  | Antisense | AUUGGUUGCAAUGCUAGUC |
| shSMIT1#1 | Sense | GCAAGUUAAAGUAAUACUA |
|  | Antisense | UAGUAUUACUUUAACUUGC |
| shSMIT1#2 | Sense | CGAUGUCACUUCCAUCUUA |
|  | Antisense | UAAGAUGGAAGUGACAUCG |

**Table S3. The primer sequences for qPCR.**

| **Gene** | **Direction** | **Sequence (5’-3’)** |
| --- | --- | --- |
| SMIT1 | Forward | AGTCTTTGGGTTGGAAT |
|  | Reverse | AATCATAAGTGTAAGTGCC |
| WTAP | Forward | ACTAAAGCAACAACAGCAGG |
|  | Reverse | CGTAAACTTCCAGGCACTC |
| YTHDF1 | Forward | CAATGAGGCTCCGTGGTC |
|  | Reverse | AAACAGCATCGTGCATAAAA |
| YTHDF2 | Forward | TTTGATGTCAGGTGGATTT |
|  | Reverse | TTGGCGTTTCTCATAGTG |
| YTHDF3 | Forward | TGCGTATGCTGGTGTCT |
|  | Reverse | GGCTTCCTCCTCTTCTT |
| MeRIP/RIP-SMIT1 | Forward | GTCTTTCTTATTGCTTCCC |
|  | Reverse | GACCCATTTAGTCAGTGTA |

**Table S4. Summary of antibody information.**

| **Name** | **Source** | **Dilution** | **Purpose** |
| --- | --- | --- | --- |
| SMIT1 | Immunoway, YT4344 | 1:1000 | primary antibody |
| cyclin D1 | ABclonal, A11022 | 1:1000 | primary antibody |
| CDK4 | ABclonal, A23522 | 1:1000 | primary antibody |
| Bcl-2 | ABclonal, A21592 | 1:1000 | primary antibody |
| Bax | ABclonal, A0207 | 1:1000 | primary antibody |
| Akt | ABclonal, A22770 | 1:1000 | primary antibody |
| p-Akt | ABclonal, AP1208 | 1:1000 | primary antibody |
| mTOR | ABclonal, A2445 | 1:2000 | primary antibody |
| p-mTOR | ABclonal, AP0115 | 1:1000 | primary antibody |
| GSK3β | ABclonal, A2081 | 1:1000 | primary antibody |
| p-GSK3β | ABclonal, AP1088 | 1:3000 | primary antibody |
| WTAP | Proteintech, 10200-1-AP | 1:8000 | primary antibody |
| YTHDF1 | Proteintech, 17479-1-AP | 1:4000 | primary antibody |
| Goat anti-rabbit | Solarbio, SE134 | 1:5000 | secondary antibody |
